# Elevated hsa_circRNA_101015, hsa_circRNA_101211, and hsa_circRNA_103470 in the Human Blood: Novel Biomarkers to Early Diagnose Acute Pancreatitis

**DOI:** 10.1155/2020/2419163

**Published:** 2020-02-18

**Authors:** Chang Liu, Xuan Zhu, Xing Niu, Lijie Chen, Chunlin Ge

**Affiliations:** ^1^Department of General Surgery, First Affiliated Hospital of China Medical University, No. 155 Nanjing North Street, Heping District, Shenyang 110001, Liaoning, China; ^2^Department of Pancreatic and Biliary Surgery, First Affiliated Hospital of China Medical University, No. 155 Nanjing North Street, Heping District, Shenyang 110001, Liaoning, China; ^3^Institute of Translational Medicine, China Medical University, No. 77 Puhe Road, Shenyang North New Area, Shenyang 110122, Liaoning, China; ^4^Department of Second Clinical College, ShengJing Hospital Affiliated to China Medical University, No. 36 Sanhao Street, Heping District, Shenyang 110004, Liaoning, China

## Abstract

**Objective:**

To explore potential biomarkers to accurately diagnose patients with acute pancreatitis (AP) at early stage and to auxiliary clinicians implement the best treatment options.

**Methods:**

We selected 3 patients with AP and 3 healthy controls for microarray analysis to obtain differentially expressed circular RNAs (circRNAs). To further verify the results of the microarray analysis, the six differentially expressed circRNAs were confirmed by quantitative polymerase chain reaction (qPCR). The diagnostic accuracy and sensitivity of differentially expressed circRNAs were assessed using the receiver operating characteristic (ROC) curve. A ceRNA network was constructed based on the 6 differentially expressed circRNAs.

**Results:**

There were 25 upregulated circRNAs and 26 downregulated circRNAs in the blood of patients with AP. Next, the qPCR verification results further confirmed three downregulated circRNAs, including hsa_circRNA_002532, has_circRNA_059665, and hsa_circRNA_104156, and three upregulated circRNAs including hsa_circRNA_101015, hsa_circRNA_101211, and hsa_circRNA_103470. Among them, hsa_circRNA_101015, hsa_circRNA_101211, and hsa_circRNA_103470 increased with the severity of the disease. ROC analysis showed that the three circRNA models show promise to diagnose AP. And a ceRNA network revealed that above six circRNAs could participate in complex regulated network.

**Conclusions:**

Elevated hsa_circRNA_101015, hsa_circRNA_101211, and hsa_circRNA_103470 could be used as novel biomarkers to diagnose AP patients.

## 1. Introduction

AP is one of the most common gastrointestinal diseases, and patients need hospitalization [1]. Although most patients are mild, about 20% of patients develop moderate or severe pancreatitis with surrounding pancreas tissue necrosis or multiple organ failure [2–4]. The overall mortality rate of AP patients is about 2%, but close to 30% in patients with persistent organ system failure. An increased risk of pancreatic cancer in AP patients was observed in a nationwide, population-based matching cohort study [5]. Furthermore, 17%–22% of AP patients have the possibility of recurrence, and 8%–16% may develop chronic pancreatitis [6, 7]. Therefore, early diagnosis is necessary for AP patients.

As a pancreatic inflammatory disease, AP has become one of the leading causes of gastrointestinal disease admission in the United States and many other countries. And the incidence rate demonstrates an increasing trend. There are many factors that cause the disease, such as gallstones, smoking, and alcohol abuse [8, 9]. Of course, about 10% of patients have no cause, and they are idiopathic pancreatitis. The patients have usually a sudden onset with severe persistent abdominal pain, and 80% of patients are accompanied by vomiting. Pain may radiate to the back, usually in the lower chest area. Therefore, it is critical to carry out some treatments within 48 hours, such as fluid resuscitation, analgesia, and nutritional support [10]. Despite the continuous improvement in diagnostic techniques, early diagnosis is still difficult for clinicians.

Therefore, it is critical to explore early diagnostic biomarkers of AP [11]. More and more evidence has confirmed that circRNAs could play an important role in many diseases. CircRNAs are produced by reverse splicing of precursor mRNAs from exons of thousands of genes in eukaryotes [12]. At the same time, circRNAs can be secreted into blood, saliva, and other body fluids as potential biomarkers for disease prediction [13]. However, the function of most circRNAs remains largely unknown. In the ceRNA mechanism, microRNAs (miRNAs) are important posttranscriptional regulators of gene expression that act on target sites in the untranslated region of messenger RNA (mRNA) by direct base pairing. The circRNA can act as a miRNA sponge to affect the activity of miRNAs in the regulation of mRNA expression [14, 15]. At the same time, circRNA abnormalities can lead to a variety of diseases. For example, the circMTO1 could inhibit the progression of hepatocellular carcinoma by promoting p21 expression, acting as a sponge of oncogenic miR-9 [16]. However, to date, no study has explored the expression of circRNAs in the blood of patients with AP. Therefore, our aim was to explore accurate biomarkers and then diagnose patients with AP as soon as possible by detecting biomarkers in the patients' blood.

## 2. Materials and Methods

### 2.1. Patient Information and Diagnostic Criteria

We reviewed 60 patients who were diagnosed with AP from April 2018 to September 2018 at the First Affiliated Hospital of China Medical University (Shenyang, China). In addition, we recruited 30 subjects who underwent routine health checks at the First Affiliated Hospital of China Medical University and showed no signs of disease as a control group. According to the “Guidelines for the diagnosis and treatment of acute pancreatitis (2014),” clinically meets 2 of the following 3 characteristics to diagnose AP: (1) abdominal pain consistent with AP; (2) serum amylase and/or lipase activity is at least 3 times higher than the upper limit of normal; (3) abdominal imaging examination is consistent with AP imaging changes.

In clinical treatment, mild acute pancreatitis (MAP) patients receive only relatively simple treatment, while severe acute pancreatitis (SAP) patients usually require intensive care. Therefore, we selected 30 MAP and 30 SAP patients to diagnose AP patients with early stage. Currently, common scoring standards are the APPACHE II scoring standard, the MCTSI scoring standard, and BISAP scoring standard. MAP diagnostic criteria were good response to fluid supplementation, without organ failure, and local or systemic complications, recovery within 1-2 weeks. And APACHE-II score <8 points or the MCTSI score <4 points or BISAP <2 are MAP. Diagnostic criteria for SAP were with persistent organ failure (48 h or more). And APACHE-II score ≥8 points or MCTSI score ≥4 points or BISAP ≥2 are SAP.

The exclusion criteria were as follows: <18 years of age, pregnant and lactating women, taking anticoagulant drugs, blood system diseases, tumors, liver disease, and gastrointestinal bleeding patients. The patients' information is shown in Table 1.

**Figure 1:**
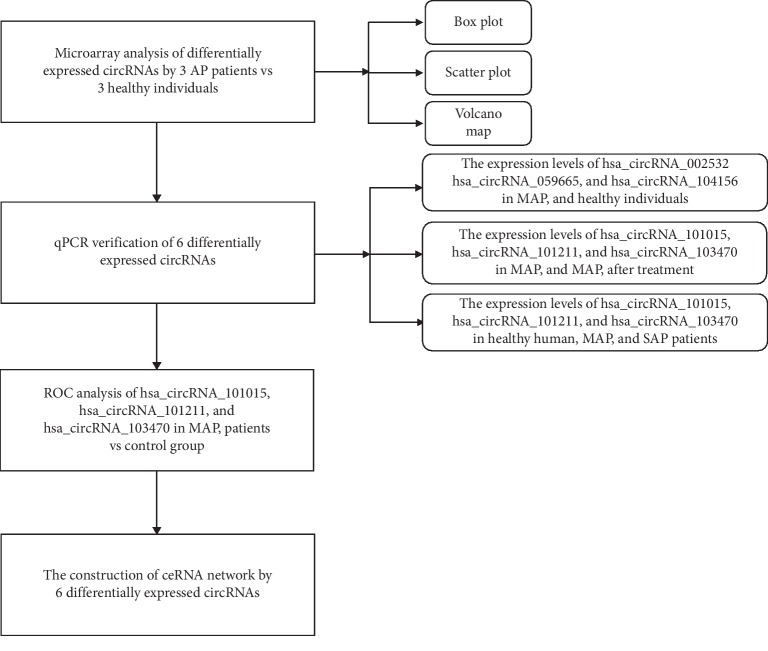
A flow chart of the experimental design.

Then we selected 3 patients with MAP and 3 healthy participants for microarray analysis. The study was approved by the Ethics Committee of the First Affiliated Hospital of China Medical University, and informed consent was obtained from all subjects.  Figure 1 depicts further experimental design details.

### 2.2. RNA Extraction and CircRNA Microarray Analysis

We collected whole blood from 3 MAP patients and 3 healthy participants. Total RNA from each sample was quantified using a NanoDrop ND-1000 (NanoDrop, Wilmington, DE, USA). Sample preparation and microarray hybridization were performed based on the standard protocol of Arraystar. Briefly, total RNA was digested with Rnase R (Epicenter, Inc.) to remove linear RNA and enrich for circular RNA. Then, the enriched circular RNA was amplified by a random priming method (Arraystar Super RNA Labeling Kit; Arraystar) and transcribed into fluorescent cRNA. The labeled cRNA was hybridized to Arraystar Human circRNA Array V2 (8 × 15 K, Arraystar). The labeled cRNA was purified by RNeasy Mini Kit (Qiagen). The concentration and specific activity of the labeled cRNA (pmol Cy3/*μ*gcRNA) were measured by NanoDrop ND-1000 (NanoDrop, Wilmington, DE, USA). 1 *μ*g of each labeled cRNA was fragmented by adding 5 *μ*l of 10× blocking agent and 1 *μ*l of 25× fragmentation buffer; then the mixture was heated at 60°C for 30 minutes, and finally 25 *μ*l of 2× hybridization buffer was added to dilute the labeled cRNA. 50 *μ*l of the hybridization solution was dispensed into a spacer slide and assembled onto a circRNA expression microarray slide. Slides were incubated for 17 hours at 65°C in Agilent Hybridization Oven. The hybridization array was fixed and scanned using an Agilent Scanner G2505C wash.

Agilent Feature Extraction software (version 11.0.1.1) was utilized to analyze the acquired array images. Quantile normalization and subsequent data processing were performed using the R software limma package (R version 3.1.2).

### 2.3. Differential Expression Analysis

A scatter plot is a visualization method used to assess circRNA expression variation. Differentially expressed circRNAs with statistical significance (FC ≥ 1.5 and *P* values ≤0.05) were identified utilizing fold change cutoffs and volcano plots, respectively. Among them, *P* value was calculated utilizing the unpaired *t*-test. Differentially expressed circRNAs between the two samples were identified by fold change filtration. Hierarchical clustering was performed to show the distinguishable circRNA expression patterns in the samples. Shanghai Kangcheng Biological Engineering Co., Ltd. of the People's Republic of China conducted microarray work.

### 2.4. qPCR Verification

**Figure 2:**
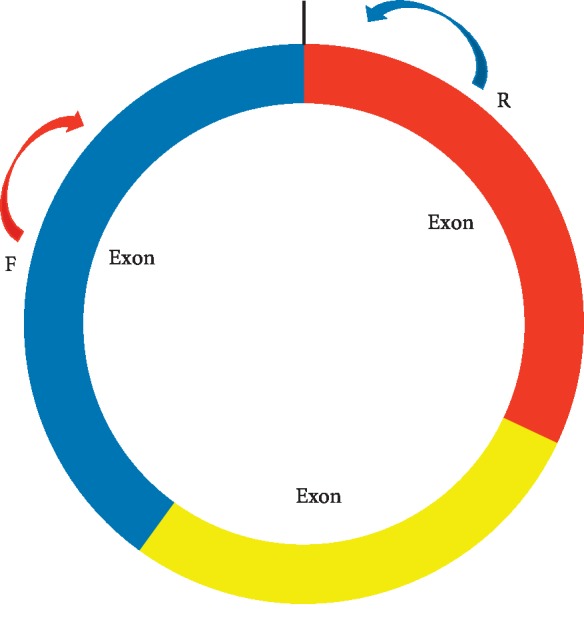
Schematic representation of polymerase chain reaction primers for specific detection of circular transcripts. Different polymerase chain reaction primers were designed for circRNA, rather than the more commonly used convergent primers.

Total RNA was extracted from the whole blood of 30 healthy participants, 30 MAP patients, and 30 SAP patients according to standard procedures. Total RNA was reverse-transcribed into cDNA kits (Roche, Penzberg, Germany) using random primers and Transcriptor First Strand cDNA Synthesis according to the manufacturer's instructions. 6 differentially expressed circRNAs were measured by qPCR using a ViiA 7 Real-time PCR System (Applied Biosystems). The reaction conditions were as follows: 95°C for 10 minutes and 40 cycles of 95°C for 10 seconds, 60°C for 60 seconds. RNA levels were normalized to human *β*-actin. All qPCR reactions were performed in triplicate. Different primers were designed for circRNAs, rather than the more commonly used convergent primers (Figure 2).  Table 2 lists all the primers.

### 2.5. Statistical Analysis

Statistical analyses were performed using SPSS (version 19.0; IBM, Armonk, NY, United States) and GraghPad Prism (version 7.0; GraphPad Software, La Jolla, CA, United States). The relative expression level of each circRNA was expressed by fold change and converted into 2^−ΔΔCt^. The expression difference in circRNAs among SAP, MAP patients, and healthy individuals and between MAP patients and posttreatment serum samples was assessed using the *t*-test. To assess the diagnostic value, ROC curve was established. The cut-off value of each circRNA was analyzed by using SPSS software. Area under the ROC curves (AUCs) were calculated to evaluate the ability of the differentially expressed circRNAs. Due to the relative small sample size for ROC analysis, the statistical power was calculated using PASS (version 15.0), under the following conditions: *α* = 0.05, AUC0 = 0.5, and *n* = 30. *P* value < 0.05 was considered statistically significant.

### 2.6. The Construction of ceRNA Network

By combining cotargeted miRNAs, we constructed a ceRNA network by cytoscape package of R language [17, 18]. In addition to measuring the number of common miRNAs, each ceRNA pair was subjected to a hypergeometric test, which was defined by four parameters: (i) *N* is the total number of miRNAs used to predict the target; (ii) *K* is the The number of miRNAs interacting with the selected gene; (iii) *n* is the number of miRNAs that interact with the candidate ceRNA of the selected gene; (iv) the miRNA number common between the two genes [19]. This test used the following formula to calculate the *P* value:

(1) $$\begin{array}{c} P=\overset{\min\left(K,n\right)}{\underset{i=c}{\sum }}\frac{\left[\begin{array}{c} K\\ i \end{array}\right]\left[\begin{array}{c} N-K\\ n-i \end{array}\right]}{\left[\begin{array}{c} N\\ n \end{array}\right]}. \end{array}$$

## 3. Results

### 3.1. Identification of Differentially Expressed CircRNAs

**Figure 3:**
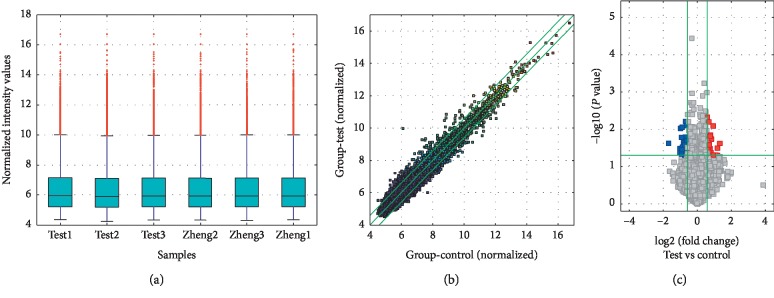
Overview of microarray features. (a) Box plots are datasets that quickly visualize circRNAs spectra (zheng: normal person's blood; test: blood of patients with acute pancreatitis). (b) Scatter plot showing circRNA expression variation between blood samples from patients with AP and matched normal persons. (c) Volcano maps of differentially expressed circRNAs.

**Figure 4:**
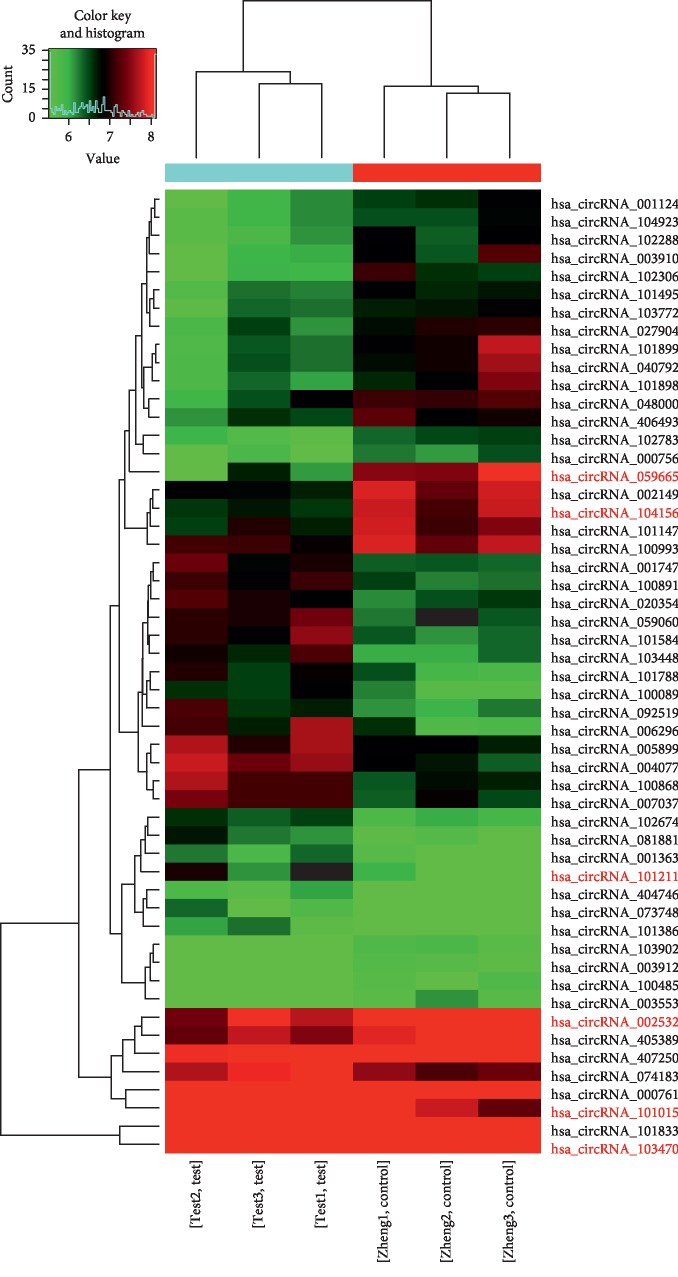
The hierarchical cluster of the differentially expressed circRNAs.

To investigate the expression profile of circRNAs in AP, we used microarray analysis to perform circRNA expression profiling in the blood of patients with MAP and matched normal humans. The box plot visualized the dataset distribution of circRNAs. After normalization, Figure 3(a) depicts that the log 2 ratios in the three pairs of samples are almost identical. At the same time, we used a scatter plot of the circRNA expression profile to evaluate the change between the two groups (Figure 3(b)), where the values of the *X* and *Y* axes are the average normalized signal values (log 2 scaling) of the sample set. And the green line is the fold line, and the circRNA above the top green line and below the bottom green line indicates that the circRNA change between the two samples is more than 1.5 times. Next, volcano maps were used to identify differentially expressed circRNAs that were statistically significant between the two groups and are shown in Figure 3(c). Among them, the vertical lines correspond to 1.5 times up and down, and the horizontal lines represent *P*=0.05. The red dot indicates upregulated circRNAs while blue represents downregulated circRNAs. We found 51 differentially expressed circRNAs in patients with MAP (FC ≥ 1.5 and *P* values ≤0.05) compared with normal subjects. Among them, Table 3 shows 25 upregulated circRNAs and 26 downregulated circRNAs are described in Table 4. Next, we clustered all the different circRNAs to characterize the expression pattern of circRNA. The results are exhibited in Figure 4. Among them, red stand for elevated and green represents downregulated circRNAs.

### 3.2. qPCR Verification

**Figure 5:**
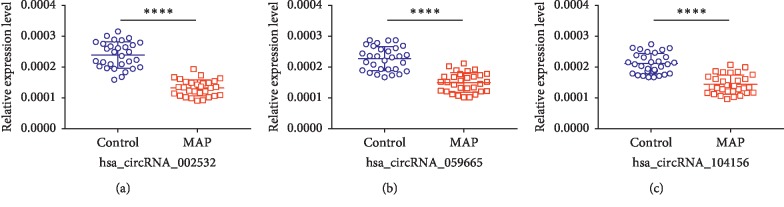
qPCR analysis of circRNA expression levels in MAP and healthy individuals including hsa_circRNA_002532, hsa_circRNA_059665, and hsa_circRNA_104156 (^*∗*^*P* < 0.05; ^*∗∗∗*^*P* < 0.001; ^*∗∗∗∗*^*P* < 0.0001).

To verify differentially expressed circRNAs in AP, we made qPCR. And we selected three upregulated circRNAs including hsa_circRNA_101015, hsa_circRNA_101211, and hsa_circRNA_103470 and three downregulated circRNAs including hsa_circRNA_002532, hsa_circRNA_059665, and hsa_circRNA_104156. We validated the expression of three downregulated circRNAs in MAP and normal humans. And the results are shown in Figure 5. *P* values are 0.008 (hsa_circRNA_002532), <0.0001 (hsa_circRNA_059665), and <0.0001(hsa_circRNA_104156), respectively.

**Figure 6:**
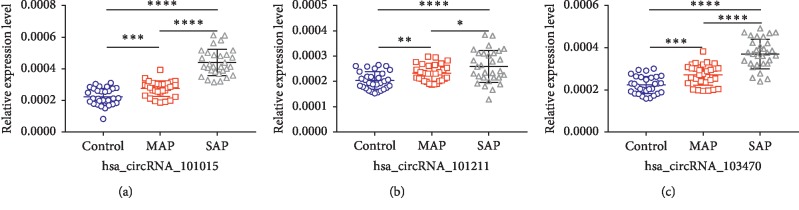
qPCR analysis of circRNA expression levels in healthy human, MAP, SAP patients including hsa_circRNA_101015, hsa_circRNA_101211, and hsa_circRNA_103470 (^*∗*^*P* < 0.05; ^*∗∗∗*^*P* < 0.001; ^*∗∗∗∗*^*P* < 0.0001).

**Figure 7:**
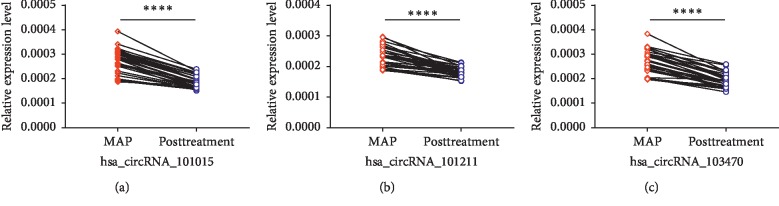
qPCR analysis of circRNA expression levels in MAP and MAP after treatment including hsa_circRNA_101015, hsa_circRNA_101211, and hsa_circRNA_103470 (^*∗*^*P* < 0.05; ^*∗∗∗*^*P* < 0.001; ^*∗∗∗∗*^*P* < 0.0001).

To further explore the relationship between the three upregulated circRNAs and disease severity, we compared the expression levels in healthy individuals, MAP, and SAP patients, respectively. As shown in Figure 6, we found that as the condition worsened, the expression levels of hsa_circRNA_101015 (*P* value was 0.0003, <0.0001, and <0.0001, respectively), hsa_circRNA_101211 (*P* value was 0.0014, 0.0478, and <0.0001, respectively), and hsa_circRNA_103470 (*P* value was 0.001, <0.0001, and <0.0001) also increased significantly. Then we validated the expression levels of three upregulated circRNAs in MAP patients and MAP after treatment. The results are demonstrated in Figure 7. After treatment, the expression levels of hsa_circRNA_101015, hsa_circRNA_101211, and hsa_circRNA_103470 in MAP were significantly declined (*P* value <0.0001).

### 3.3. ROC Analysis of hsa_circRNA_101015, hsa_circRNA_101211, and hsa_circRNA_103470 in Patients with AP

**Figure 8:**
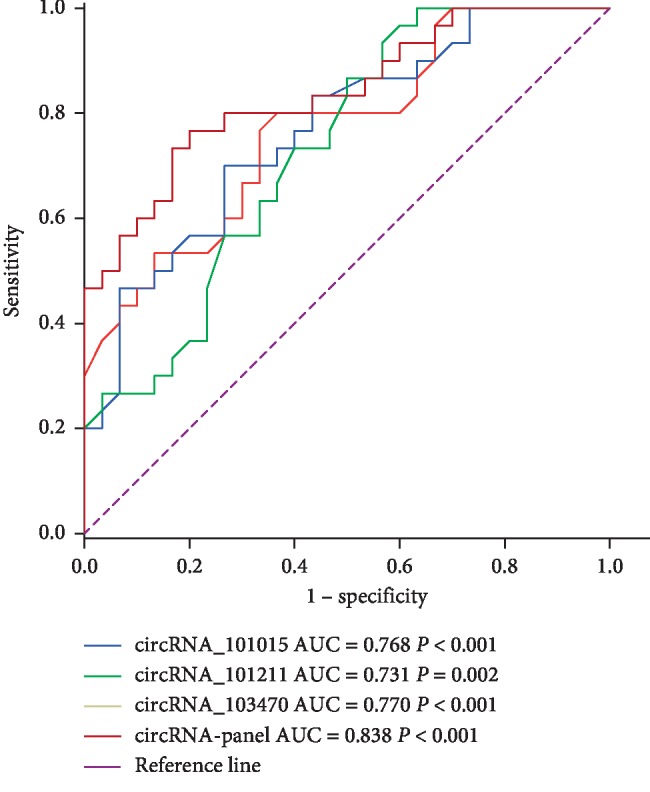
ROC analysis of hsa_circRNA_101015, hsa_circRNA_101211,and hsa_circRNA_103470 in MAP patients versus control group.

The ROC curve was constructed to assess the diagnostic significance of the three elevated circRNAs (Figure 8). RUCs of hsa_circRNA_101015, hsa_circRNA_101211, and hsa_circRNA_103470 MAP patients with healthy people were 0.768 (95% CI, 0.651–0.886, *P* < 0.001, power = 0.97119), 0.731 (95% CI, 0.605–0.857, *P*=0.002, power = 0.90168), 0.770 (95% CI, 0.653–0.887, *P* < 0.001, power = 0.97340), respectively. RUC of the three circRNAs combination was 0.838 (95% CI, 0.738–0.937, *P* < 0.001, power = 0.99954). The above results show that hsa_circRNA_101015, hsa_circRNA_101211, and hsa_circRNA_103470 can be used as biomarkers to diagnose AP at early stage.

### 3.4. The Construction of ceRNA Network

**Figure 9:**
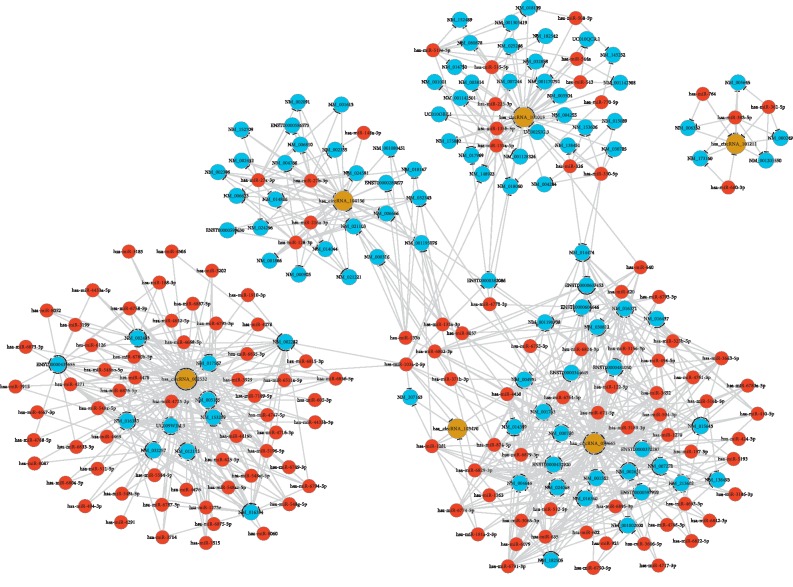
The ceRNA network of 6 differentially expressed circRNAs including hsa_circRNA_101015; hsa_circRNA_103470; hsa_circRNA_059665; hsa_circRNA_002532; hsa_circRNA_101211; hsa_circRNA_104156.

Recent evidence suggests that circular RNA plays a crucial role in the regulation of miRNA-mediated gene expression regulation by isolating miRNAs. Their interaction with disease-associated miRNAs suggests that circular RNA is important for disease regulation [20]. To assess the potential function of circRNA, we investigated potential miRNAs that bind to circRNA. Then we constructed a ceRNA network for the six differentially expressed circRNAs. In the ceRNA network, there are 241 nodes and 831 edges (Figure 9). Among them, for all nodes, red represents microRNAs, light-blue color represents protein_coding RNAs, and at the same time, brown color represents circular RNAs. Considering all edges, T-shape arrow represents directed relationships, while edges without arrow stand for undirected relationships. Specific details are shown in Supplementary [Supplementary-material supplementary-material-1].

## 4. Discussion

AP is an inflammatory process of the pancreas and has become an increasingly common clinical disease. In the second or third week of the disease, 40–70% of patients develop infectious necrosis and are the leading cause of late death [21]. In the United States, AP accounts for $2.5 billion in medical expenses, and the number of hospital admissions is about 275,000 per year [22]. Because of the high mortality rate of 20–30% in severe cases of AP, early detection of patients who may need to be transferred to the intensive care unit (ICU) is critical. For patients with AP, accurate diagnosis, appropriate triage, high-quality supportive care, monitoring and treatment of complications, and prevention of recurrence are also critical [23]. The evaluation of biomarkers helps to further improve the identification of high-risk patients. In general, the clinical treatment decisions for AP depend primarily on the severity of the condition. MAP is usually self-limiting and does not cause death. However, SAP can develop rapidly, leading to multiple organ failure and becoming life threatening. Therefore, the treatment of MAP patients is relatively simple and requires only a short hospitalization, while the treatment of SAP patients usually involves intensive care. Whether it is MAP or SAP, quick and accurate diagnosis is still very difficult.

Accurate diagnosis of AP may allow for effective treatment to begin earlier. At present, the accuracy of different scoring systems is not high, and a unique model is needed [24]. There are several laboratory tests such as blood urea nitrogen, creatinine, and hematocrit [25]. However, there is virtually no laboratory test that consistently and accurately predicts the severity of AP earlier [26, 27]. Many predictive systems use CT findings, but CT evidence of SAP lags behind clinical findings, and early CT studies may underestimate the severity of the disease. The scoring system is complex and cumbersome; therefore, these scoring systems are not a substitute for the clinician's experience assessment.

In recent years, circRNA has received wide attention as a new class of endogenous and regulatory noncoding RNAs. At the same time, with the widespread use of RNA sequencing (RNA-seq) technology and bioinformatics prediction, a large number of circRNAs have been identified. So far, no study has examined the role of circRNA in AP. Considering that circRNA is involved in a variety of diseases, it is necessary to explore differences in the expression of circRNA in patients with AP. Microarrays are an effective tool for analyzing circRNA. Therefore, in our study, we made full use of microarray technology and selected three patients with MAP and three healthy individuals. Our aim was to explore the differential expression of circRNA in the patients' blood with AP to diagnose AP patients as soon as possible. As a result, we found 25 upregulated circRNAs and 26 downregulated circRNAs. The vast majority of circRNAs have not been studied and require more in-depth exploration.

To further validate the six circRNAs in AP, we performed qPCR analysis. The results of qPCR studies provide novel biomarkers for molecular diagnosis and evaluation in AP. Three circRNAs including hsa_circRNA_002532 hsa_circRNA_059665, and hsa_circRNA_104156 were downregulated in MAP compared with healthy individuals. Three upregulated circRNAs including hsa_circRNA_101015, hsa_circRNA_101211, and hsa_circRNA_103470 significantly increased expression levels as the condition worsens. Furthermore, the expression levels of hsa_circRNA_101015, hsa_circRNA_101211, and hsa_circRNA_103470 were significantly reduced after treatment. This result suggests that the above three circRNAs may be involved in the development of AP and show promise as potential biomarkers for AP. To evaluate the specificity and sensitivity of the hsa_circRNA_101015, hsa_circRNA_101211, and hsa_circRNA_103470, we performed a ROC curve for further validation. The RUC was 0.768, 0.731, and 0.770, respectively, and the combined RUC values of the three circRNAs were 0.838. Therefore, the three circRNAs can be used as diagnostic biomarkers for AP with high sensitivity.

However, the pathogenesis of AP remains unknown. Therefore, the 6 differentially expressed circRNAs were selected to construct a ceRNA network including three upregulated hsa_circRNA_101015, hsa_circRNA_101211, and hsa_circRNA_103470 and three downregulated hsa_circRNA_002532 hsa_circRNA_059665, and hsa_circRNA_104156. In the ceRNA network, we found a complex regulatory network for the above six circRNAs. However, the above six differentially expressed circRNAs excluding hsa_circRNA_104156 have not been reported. Considering that circRNAs can act as sponges of miRNAs, it is of importance to explore miRNAs in AP. More importantly, miRNAs play a critical role in the progression of AP. For example, a previous study found that miR-92b, miR-10a, and miR-7 are downregulated in the blood of patients with AP and can be used to distinguish between patients with AP and healthy cases. In addition, the expression level of miR-551b-5p distinguishes between MAP and SAP [28]. However, these six circRNAs cannot interact with above these miRNAs. In the ceRNA network, hsa_circRNA_101015 could become sponges of several miRNAs such as hsa-miR-135a/b and hsa-miR-543. It has been reported that hsa-miR-135 was upregulated in the serum of mice with acute pancreatitis [29]. Furthermore, it has reported that hsa-miR-135a/b binding polymorphism can reduce the expression of CD133 and reduce the risk of lung cancer and improve the prognosis of patients [30]. Abnormalities in hsa-miR-543 can lead to increased proliferation of multiple cancers, such as esophageal cancer, prostate cancer, and osteosarcoma [31–33]. In the ceRNA network, hsa-miR-135 could be a target of hsa_circRNA_101015. It has been found that hsa-miR-216a was downregulated in the serum of mice with acute pancreatitis [34]. Elevated serum hsa-miR-122 has been considered as a noninvasive marker for acute pancreatitis, which could become a target miRNA of hsa_circRNA_059665 as shown in the ceRNA network [35]. Moreover, hsa-miR-148a has been validated to be lowly expressed in acute pancreatitis [36]. Our results showed that hsa-miR-148a could be a potential target miRNA of hsa_circRNA_059665. Furthermore, hsa-miR-148a could inhibit autophagy through IL-6/STAT3 axis in AP in acute pancreatitis. According to the ceRNA network, hsa_circRNA_104156 could be considered as a sponge of hsa-miR-148a.

Therefore, we inferred that the six circRNAs could participate in pathogenic process of AP. However, specific research mechanisms need to be explored. In the future, we would continue to research the molecular mechanisms of hsa_circRNA_101015, hsa_circRNA_101211, and hsa_circRNA_103470 in AP.

## 5. Conclusion

After microarray analysis and qPCR, we found three downregulated circRNAs including hsa_circRNA_002532, hsa_circRNA_059665, and hsa_circRNA_104156 and three upregulated circRNAs including hsa_circRNA_101015, hsa_circRNA_101211, and hsa_circRNA_103470 in the blood of AP patients. The ceRNA network revealed that the six circRNAs could play a critical role in AP. Therefore, elevated hsa_circRNA_101015, hsa_circRNA_101211, and hsa_circRNA_103470 can be used as biomarkers in the blood to diagnose AP at early stage. The molecular mechanisms of elevated hsa_circRNA_101015, hsa_circRNA_101211, and hsa_circRNA_103470 in the pathological processes of AP would be explored in our further experiments.

## Figures and Tables

**Table 1:** Demographic and clinical characteristics of the MAP patients, SAP patients, and the control group.

Characteristic	Control (*n* = 30)	MAP (*n* = 30)	SAP (*n* = 30)	*P* ^a^	*P* ^b^
Age (y)	48.01 ± 12.67	48.26 ± 15.24	49.21 ± 10.32		
F/M (*n*)	15/15	12/18	13/17		
MCTSI		1.4 ± 0.93	4.98 ± 1.04	<0.001	<0.001
apache-ii		3.09 ± 2.94	9.87 ± 4.01	<0.001	<0.001
BISAP		0.91 ± 0.69	2.64 ± 1.01	<0.001	<0.001

^a^SAP versus control; ^b^SAP versus MAP.

**Table 2:** Primers for circRNA and mRNA levels in qPCR.

Target ID	Primer sequence, 5′-3′	Product size in bp
*β*-actin (human)	F: 5′GTGGCCGAGGACTTTGATTG 3′ R: 5′ CCTGTAACAACGCATCTCATATT 3′	73
hsa_circRNA_002532	F: 5′TGGGAGTTTTCTGCTGATGAT 3′ R: 5′ GGGTTTCTTTCTCATCTCTCTCA 3′	119
hsa_circRNA_101015	F: 5′ TATTGCCTTAGATCCTTCAAGTG 3′ R: 5′ TAGCATCAACCAATCGCAAGT 3′	78
hsa_circRNA_101211	F: 5′ TGGTGGACGCATTTTCAGC 3′ R: 5′ TCAGCACTTTGGTTAATCTTTCA 3′	87
hsa_circRNA_103470	F: 5′ TAACACGCTGGCCCATTACAAG 3′ R: 5′ GTCCAGGTCCCGAAGGATGTAG 3′	69
hsa_circRNA_059665	F: 5′ AGACGCCTCCAGATGCCCTT 3′ R: 5′ ACCAGACTGCAGGGACGGTGT 3′	103
hsa_circRNA_104156	F: 5′ AGAACTTCCTCGCACTGTGA 3′ R: 5′ GGGAGCTATGTGAACGAACG 3′	83

**Table 3:** 25 upregulated in patients with AP (FDR, false discover rate; FC, fold change).

CircRNA	Alias	*P* value	FDR	FC (abs)	Chrom	best_transcript	Gene symbol
hsa_circRNA_001747	hsa_circ_0000246	0.014721747	0.999852393	1.5883102	chr10	uc009xqp.1	MCU
hsa_circRNA_000761	hsa_circ_0000761	0.032659244	0.999852393	1.6521444	chr17	NM_032875	FBXL20
hsa_circRNA_092519	hsa_circ_0001233	0.031884015	0.999852393	1.6043929	chr22	NM_020831	MKL1
hsa_circRNA_102674	hsa_circ_0053932	0.004725614	0.999852393	1.5060131	chr2	NM_000627	LTBP1
hsa_circRNA_020354	hsa_circ_0020354	0.016055616	0.999852393	1.5389787	chr10	NM_001329	CTBP2
hsa_circRNA_059060	hsa_circ_0059060	0.020943584	0.999852393	1.6047327	chr2	NM_004404	SEPT2
hsa_circRNA_074183	hsa_circ_0074183	0.038344155	0.999852393	1.5310808	chr5	NM_199189	MATR3
hsa_circRNA_101015	hsa_circ_0000378	0.023899101	0.999852393	2.5012237	chr12	NM_002336	LRP6
hsa_circRNA_100089	hsa_circ_0010501	0.017554998	0.999852393	1.7280045	chr1	NM_001397	ECE1
hsa_circRNA_101386	hsa_circ_0004008	0.033240163	0.999852393	1.6859948	chr14	NM_014982	PCNX
hsa_circRNA_101788	hsa_circ_0038872	0.036463254	0.999852393	1.68241	chr16	NM_004320	ATP2A1
hsa_circRNA_103470	hsa_circ_0067301	0.049994992	0.999852393	2.2660602	chr3	NM_015103	PLXND1
hsa_circRNA_006296	hsa_circ_0006296	0.049042374	0.999852393	2.11451	chr1	NM_004284	CHD1L
hsa_circRNA_073748	hsa_circ_0073748	0.04252091	0.999852393	1.7548801	chr5	NM_005573	LMNB1
hsa_circRNA_100891	hsa_circ_0023685	0.006324353	0.999852393	1.6050939	chr11	NM_002576	PAK1
hsa_circRNA_404746		0.036733957	0.999852393	1.5850621	chr10	NM_018590	CSGALNACT2
hsa_circRNA_005899	hsa_circ_0005899	0.040331215	0.999852393	1.5983183	chr1	NM_000081	LYST
hsa_circRNA_101584	hsa_circ_0036200	0.018409954	0.999852393	1.7862666	chr15	NM_002654	PKM
hsa_circRNA_103448	hsa_circ_0067029	0.014194703	0.999852393	1.7205437	chr3	NM_006810	PDIA5
hsa_circRNA_004077	hsa_circ_0004077	0.008137346	0.999852393	1.9160136	chr16	NM_020927	VAT1L
hsa_circRNA_001363	hsa_circ_0000172	0.031341076	0.999852393	1.5314787	chr1	ENST00000340006	CSRP1
hsa_circRNA_081881	hsa_circ_0081881	0.015325331	0.999852393	1.6956771	chr7	NM_002736	PRKAR2B
hsa_circRNA_007037	hsa_circ_0007037	0.023032396	0.999852393	1.5823312	chr9	NM_024617	ZCCHC6
hsa_circRNA_101211	hsa_circ_0029407	0.03202411	0.999852393	2.2688595	chr12	uc001uhy.1	GLT1D1
hsa_circRNA_101833	hsa_circ_0039908	0.04884264	0.999852393	1.9258456	chr16	NM_017803	DUS2
hsa_circRNA_100868	hsa_circ_0023255	0.026079049	0.999852393	1.6328345	chr11	NM_001876	CPT1A

**Table 4:** 26 downregulated circRNAs in patients with AP (FDR, false discover rate; FC, fold change).

CircRNA	Alias	*P* value	FDR	FC (abs)	Chrom	best_transcript	Gene symbol
hsa_circRNA_102783	hsa_circ_0007532	0.006107722	0.999852393	1.6128104	chr2	NM_015341	NCAPH
hsa_circRNA_101147	hsa_circ_0028255	0.04131669	0.999852393	1.6696469	chr12	NM_002973	ATXN2
hsa_circRNA_407250		0.047617993	0.999852393	1.668651	chr9	NM_004269	MED27
hsa_circRNA_104923	hsa_circ_0002303	0.029109485	0.999852393	1.545763	chr9	NM_001006617	MAPKAP1
hsa_circRNA_003912	hsa_circ_0003912	0.007972547	0.999852393	1.5662106	chr19	NM_001352	DBP
hsa_circRNA_406493		0.035076882	0.999852393	1.5155585	chr4	ENST00000508171	LIN54
hsa_circRNA_104156	hsa_circ_0001626	0.009445502	0.999852393	1.9847946	chr6	NM_021813	BACH2
hsa_circRNA_100485	hsa_circ_0006635	0.030459921	0.999852393	1.5844839	chr1	NM_014801	PCNXL2
hsa_circRNA_102288	hsa_circ_0046702	0.022518826	0.999852393	1.7462888	chr18	NM_005433	YES1
hsa_circRNA_003910	hsa_circ_0003910	0.032896633	0.999852393	2.0304476	chr3	ENST00000470751	SUMF1
hsa_circRNA_000756	hsa_circ_0000756	0.024633983	0.999852393	1.7492495	chr17	NM_024857	ATAD5
hsa_circRNA_059665	hsa_circ_0059665	0.02380009	0.999852393	3.2119391	chr20	NM_015600	ABHD12
hsa_circRNA_103902	hsa_circ_0006916	0.016212163	0.999852393	1.637866	chr5	NM_004272	HOMER1
hsa_circRNA_101495	hsa_circ_0003838	0.025961386	0.999852393	1.5569843	chr15	NM_173500	TTBK2
hsa_circRNA_002149	hsa_circ_0001627	0.008793528	0.999852393	1.8600357	chr6	ENST00000343122	BACH2
hsa_circRNA_405389		0.02592997	0.999852393	1.5178686	chr15	NM_004255	COX5A
hsa_circRNA_101899	hsa_circ_0000724	0.042899134	0.999852393	2.0353244	chr16	NM_017566	KLHDC4
hsa_circRNA_103772	hsa_circ_0008910	0.048867847	0.999852393	1.6283164	chr4	NM_001921	DCTD
hsa_circRNA_001124	hsa_circ_0001124	0.023465588	0.999852393	1.7343183	chr2	NM_032329	ING5
hsa_circRNA_003553	hsa_circ_0003553	0.020016881	0.999852393	1.5533047	chr1	NM_013441	RCAN3
hsa_circRNA_002532	hsa_circ_0002532	0.029871425	0.999852393	1.8331005	chrX	TCONS_00017187	XLOC_008000
hsa_circRNA_102306	hsa_circ_0046995	0.016459014	0.999852393	1.9022111	chr18	NM_032142	CEP192
hsa_circRNA_048000	hsa_circ_0048000	0.04965674	0.999852393	1.6658957	chr18	NM_198531	ATP9B
hsa_circRNA_027904	hsa_circ_0027904	0.02618334	0.999852393	1.6835986	chr12	NM_020244	CHPT1
hsa_circRNA_101898	hsa_circ_0040786	0.035050956	0.999852393	1.8735586	chr16	NM_017566	KLHDC4
hsa_circRNA_100993	hsa_circ_0002484	0.032886032	0.999852393	1.519562	chr11	NM_014155	ZBTB44
hsa_circRNA_040792	hsa_circ_0040792	0.046578567	0.999852393	1.8944367	chr16	NM_017566	KLHDC4

## Data Availability

The data used to support the findings of this study are included within the supplementary information file.

## Abbreviations

AP: Acute pancreatitis
circRNAs: Circular RNAs
qPCR: Quantitative polymerase chain reaction
ROC: Receiver operating characteristic
SAP: Severe acute pancreatitis
MAP: Mild acute pancreatitis
miRNAs: MicroRNAs
AUCs: Area under the ROC curves.

## Supplementary Materials

Supplementary Materials Supplementary Table 1: the details of the ceRNA network.
